# mtDNA from the Early Bronze Age to the Roman Period Suggests a Genetic Link between the Indian Subcontinent and Mesopotamian Cradle of Civilization

**DOI:** 10.1371/journal.pone.0073682

**Published:** 2013-09-11

**Authors:** Henryk W. Witas, Jacek Tomczyk, Krystyna Jędrychowska-Dańska, Gyaneshwer Chaubey, Tomasz Płoszaj

**Affiliations:** 1 Department of Molecular Biology, Medical University of Łódź, Łódź, Poland; 2 Department of Anthropology, Cardinal Stefan Wyszyński University, Warszawa, Poland; 3 Estonian Biocenter, Tartu, Estonia; Natural History Museum of Denmark, Denmark

## Abstract

Ancient DNA methodology was applied to analyse sequences extracted from freshly unearthed remains (teeth) of 4 individuals deeply deposited in slightly alkaline soil of the Tell Ashara (ancient Terqa) and Tell Masaikh (ancient Kar-Assurnasirpal) Syrian archaeological sites, both in the middle Euphrates valley. Dated to the period between 2.5 Kyrs BC and 0.5 Kyrs AD the studied individuals carried mtDNA haplotypes corresponding to the M4b1, M49 and/or M61 haplogroups, which are believed to have arisen in the area of the Indian subcontinent during the Upper Paleolithic and are absent in people living today in Syria. However, they are present in people inhabiting today’s Tibet, Himalayas, India and Pakistan. We anticipate that the analysed remains from Mesopotamia belonged to people with genetic affinity to the Indian subcontinent since the distribution of identified ancient haplotypes indicates solid link with populations from the region of South Asia-Tibet (Trans-Himalaya). They may have been descendants of migrants from much earlier times, spreading the clades of the macrohaplogroup M throughout Eurasia and founding regional Mesopotamian groups like that of Terqa or just merchants moving along trade routes passing near or through the region. None of the successfully identified nuclear alleles turned out to be ΔF508 CFTR, LCT-13910T or Δ32 CCR5.

## Introduction

The still ongoing debate on the origin of people inhabiting ancient Mesopotamia during the long history of the region [1] has encouraged the authors to attempt an isolation and analysis of mtDNA sequences, which, if available, can deliver information of primary significance. Although they do not allow the details regarding the life of the individual to be reconstructed, DNA analysis provides important insight into his/her ancestry. Fossil sequences are preferably isolated from remains unearthed in permafrost or temperate regions, and only rarely from skeletal material found in a subtropical arid climate, probably due to the widespread belief that access to amplifiable sequences is highly limited in such cases. Thus, only scarce data from the Mesopotamia region are available [2], [3]. However, using ancient DNA methodology, we aimed to confirm the possibility of isolating amplifiable sequences from the skeletons staying under conditions favourable for DNA survival. Having access to skeletal material in the case of one of the studied specimens we analysed both mtDNA and nuDNA sequences. Three others were analysed only to confirm their origin on the basis of HVR-I sequence. Studied remains were excavated at two archaeological sites in the middle Euphrates valley and dated between the Early Bronze Age and the Late Roman period. The obtained data enrich the as yet modest database of Mesopotamian ancient DNA and suggest a possible genetic link of the region with the Indian subcontinent in the past leaving no traces in the modern population.

## Materials and Methods

The studied skeletal material is now a part of a collection deposited in the anthropological museum located at the excavation base in Tell Ashara, and labeled by the numbers used in the paper. All necessary permits from Dept. of Archaeology and Museology, Ministry of Culture, Arabian Republic of Syria, were obtained for the needs of described study, which complied with all relevant regulations.

### Skeletal Material

Human remains, after careful mechanical cleaning, were subjected to anthropological analysis by J.T. according to the Standards for Data Collection from Human Skeletal Remains [4]. Sex was determined basing on the Phenice method and morphology of the skull (cf. [4]). Biological age was estimated using morphology changes within pubic symphysis [5] and standards for topography changes of auricular surface (cf. [4], [6]). To confirm biological age cranial suture closure, epiphyseal closure [7] and surface wear scoring systems for the anterior [8] and posterior teeth [9] were used.

After extraction from the mandible, in sterile conditions, each tooth was transferred to separate small container and frozen at −28°C. At this stage J.T. was the only person who came into contact with the remains after unearthing.

Below characterized are the specimens which delivered amplifiable DNA sequences. Their age was estimated on the basis of stratigraphy and grave equipment. MK – Tell Masaikh; TQ – Terqa.

Specimen MK 11G 107, excavated at the Tell Masaikh site during the 2006 excavation season (male, age ∼30). Pathological changes within the skull and postcranial bones were found, but not recognized as specific markers resulting from inflammation, local viral or bacterial infections or generalized chronic lesions. The suggested cause of the changes was more a malfunction of the haematopoiesis process, not excluding thalassemia [10], [11]. Grave deposits (e.g. jar) and the east-west orientation of the grave indicated the turn of the Late Roman and Islamic periods as the time of burial (500–700 AD) located under the floor of a Roman house [12]. Molecular analysis was performed on DNA isolated from 3 premolars (FDI: 44, 45, 15) and an upper molar (FDI: 18).

Specimen MK 13G 117, excavated at the Tell Masaikh site during the 2008 season (female, age 25–26), together with some ceramic sherds and bronze jewellery originating from the Late Roman period (200–300 AD), allowing for terminus post quem dating of the skeleton [9]. A neurocranium with a fragment of postcranial skeleton was found among the preserved bones. Molecular analysis was performed on DNA isolated from a lower molar (FDI: 46) and an upper premolar (FDI: 24).

Specimen TQ 28F 112, excavated at the Terqa site during the 2008 season (male, age 40–44). The grave was dated to the Early Bronze Age, stage IV.0/IV.1 (2650-2450 BC), on the basis of chronology of the tomb, stratigraphy of the site and the equipment found. Molecular analysis was performed on DNA isolated from a lower second molar (FDI: 47) and a lower premolar (FDI: 35). In the case of this specimen the obtained age, 2650-2450 BC, closely matched the radiocarbon dating of the male skeleton TQ 26F 222 unearthed two years earlier and found together with the remains of an onager (Equus hemionus) ∼3 m from TQ 28F 112 within the same level: 2581 (93.9%) 2338 cal. BC, calendar age 3970±45 C14 BP.

Specimen TQ 28F 256, a skeleton excavated at the Terqa site during the 2008 season (male, age 25–29). Dating of the tomb indicated a burial time in the middle Bronze Age, stage III.1 (2200–1900 BC). Tomb chronology was based on the type of artefacts found together with the skeleton and stratigraphy of the site which were comparable to others found in Terqa. Molecular analysis was performed on DNA isolated from 2 upper molars (FDI: 26, 28).

### Indirect Assessment of DNA Persistence

CaCO_3_ content was determined in six soil samples from the Tell Masiakh site –700 g each - by the Scheibler method (1 g of sample and 5 ml of 10% HCl). The pH of the supernatant was measured in 10 g of soil mixed with 25 ml of distilled water after 24 hrs. Collagen content was determined after incubating 300 mg of toothpowder in 5 ml 1 M HCl at 48°C for 5 hrs., followed by desiccation of the insoluble fraction at 56°C for 18 hrs., and a few washings, until a neutral pH was obtained.

### Isolation and Analysis of DNA

DNA was isolated according to commonly accepted precautions [2], [3], . Mechanical cleaning (Dremel®) was followed by washing in NaClO for 30 min., rinsing in 96% ethanol and exposure to UV light for 30 min. on each side. Each tooth was then ground in a freezer mill (SPEX SamplePrep 6770). Tooth powder, after decalcification for 48 hrs. (0.5 and 0.9 g in 0.5 M EDTA, pH = 8.0), was incubated with proteinase K and *N*-phenacyltiazolium bromide (PTB) at 56°C for the next 2 hrs. DNA was isolated in a semi-automated, closed system – MagNA Pure® Compact Nucleic Acid Purification System (Roche) – according to the manufacturer’s recommendations and quantified (Qubit 2.0, Invitrogen). PCR of isolated sequences was completed within 24 hrs. Reaction was performed in the final volume of 25 µl, including 2–4 µl of the sample extract and standard reagents including Taq Gold ® (Applied Biosystems), for 38 cycles at different annealing temperature for HVR-I, HVR-II, coding region, *HbA1*, *HbA2*, *HBB*, *CFTR* and *MCM6* and *CCR5*, which are listed together with primer sequences in the table S1. Amplicons were sequenced and alleles identified according to methodology described by Płoszaj et al. [16]. Wild and mutated (*ΔF508*) *CFTR* alleles were amplified with KAPA HRM Fast PCR Kit (Kapa Biosystems). A 3-bp difference between pathological and physiological alleles was followed by HRM (High Resolution Melting) method on Eco Real-Time PCR (Illumina). *Δ32 CCR5*/*CCR5* alleles was identified in10% polyacrylamide gel using silver staining.

### Authentication of DNA Sequences

**Figure 1:**
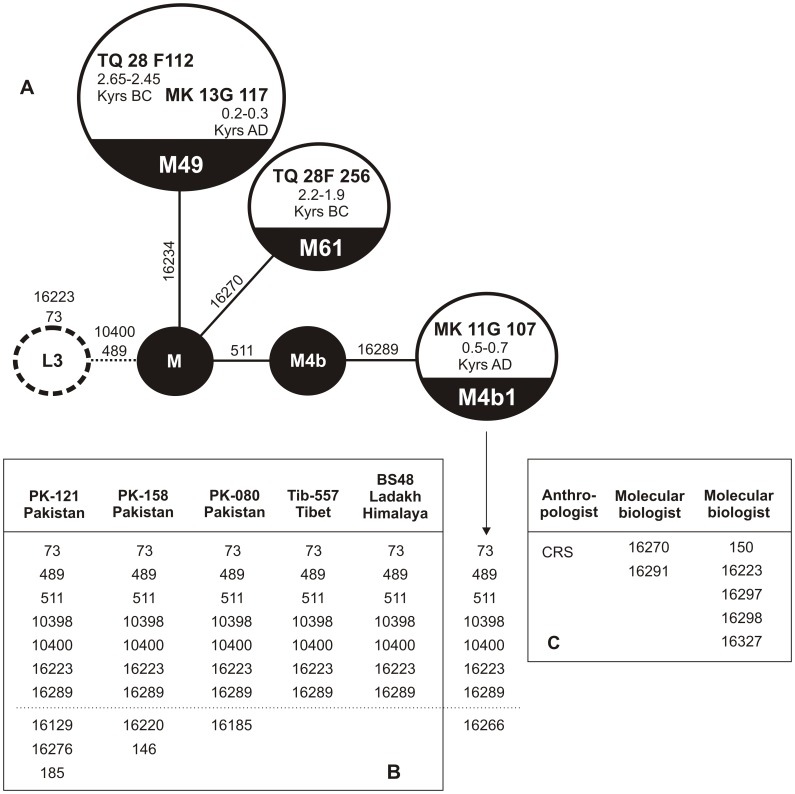
Phylogenetic position and mutation differences identified in studied specimens and retrieved in moderns. **A.** Four specimens unearthed at Tell Masaikh and Tell Ashara archaeological sites in the middle Euphrates valley. **B.** Haplotypes found in people living today in Tibet [23], Himalayas [24] and Pakistan [25] lacking 16311 mutation (reverse mutation) as observed in MK 11G 107. **C.** Haplotype profiles of scientific staff involved in collection of skeletal material, DNA isolation and its analysis. Mutation differences are shown using revised Cambridge Reference Sequence (rCRS) [38].

**Figure 2:**
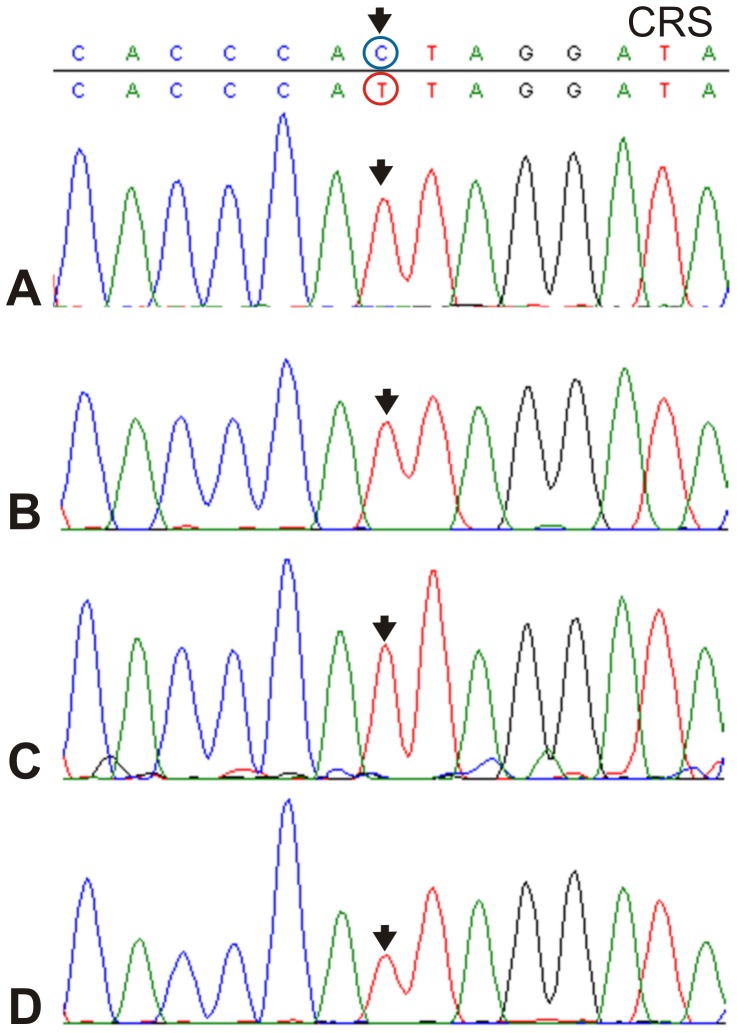
16270 change found in individual TQ 28F 256 and identified in four separate runs of PCR product obtained from different portions of pulverized different teeth. **A.** Upper molar FDI 26, preparation 1; **B.** Upper molar FDI 26, preparation 2; **C.** Upper molar FDI 28, preparation 1; **D.** Upper molar FDI 28, preparation 2.

Isolation and molecular analysis were carried out in an ancient DNA lab where molecular analysis of modern molecules has never been conducted. Powdering, extraction and amplification of DNA were carried out by lab staff wearing disposable protective clothes (gloves, face masks and laboratory coats). To avoid contamination, commonly used precautions were applied. A laminar flow hood (Heraeus Biohazard II) and DNA-free disposables equipped with filters (Sarstedt) provided appropriate working conditions. Permanent decontamination of instruments and the lab surface with DNA-ExitusPlus™ solution (AppliChem) after each experiment was followed by UV irradiation until the next activity. Independent extractions of DNA from more than one tooth of each individual were completed and verified based on the result of multiple mock controls amplification. Each tooth was powdered independently by a different staff member of known genotype registered in our Personal Genetic Identification Database (PGID). All the lab members as well as co-operating anthropologists and archaeologists are represented by haplogroups with easily recognizable mutations which are characteristic for the individual and present with those identifying the haplogroup, like 16126 or 16188 with hg H, 16297 with hg C, 16189 with hg U5, 16284 and 16319 with hg K, and so on. Besides the full mtDNA haplotypes also *MCM6*, *CFTR* and *CCR5* genotypes enrich the PGID. This allows contaminating DNA from lab staff to be easily identified, in case it is present in the analysed sample. The above procedure, a common practice in our laboratory was, crucial for the authentication of Syrian samples since only 3 persons were involved in their processing since unearthing at the archaeological sites, i.e. the anthropologist digging out and examining skeletal material and two molecular biologists whose mtDNA haplotypes (Fig. 1C) and nuDNA alleles were identified (Tab. S2). Difference between obtained mtDNA sequences (clades of haplogroup M) and that of people involved in the archaeological/molecular procedures (CRS, C and U5, Table S1) as well as those carried by modern Syrians (hg M is absent in the region) is one of the significant indicators of the authenticity of isolated DNA. Moreover, the presence of nuDNA alleles, in particular LCT-13910T and *CCR5*, in MK 11 G107 and people involved in the analysis confirms the authenticity of isolated fragments (Table S2). Considering the fact that possible contamination routes can be followed (3 people involved) and also that teeth, as the most resistant to DNA contamination fragment of skeleton [17], were used for the analysis, we have chosen the methodology of direct sequencing of amplicons from multiple isolates from the same individual as described by Winters et al. [14]. The method was applied instead of time and cost consuming cloning as a sufficient one for the data authentication. At least two isolates from each of two different teeth of each individual were sequenced. Additional isolates were not necessary since the profiles of multiple sequenograms exhibited consensus sequencing result, thus confirming the authenticity of observed changes within analysed sequences (Fig. 2).

## Results and Discussion

Two factors suggested the possibility of successful isolation of authentic DNA sequences prior to the procedure, indicating that the remains were deposited in favourable conditions. Firstly, the slightly alkaline soil at a pH of 8.1 in samples from the New Assyrian and the Islamic levels contained 16.3% and 18.9% of CaCO_3_ respectively. Secondly, the depth at which remains were deposited (1.5–2.0 m below the surface) suggested rather moderate than high temperature of the remains’ surroundings. Both chemical and physical parameters resulted probably in preservation of collagen content amounting to 3.41% [18] in the tooth of specimen MK 11G107.

The present study was undertaken mostly as an attempt to confirm the origin of individual MK 11G107 living 1.3–1.5 Kyrs ago whose remains were found in Tell Masaikh. Anthropological examination of the skeleton revealed features that might be recognized as signs of anaemia, and probably thalassemia [19], which in turn might suggest him having been a newcomer, e.g. from the neighbouring Mediterranean region.

### mtDNA Sequence

Changes found within the HVR-I sequence of specimen MK 11G107 indicated the presence of the M4b1 haplogroup according to HaploGrep [20] (M65a by the earlier nomenclature and PhyloTree). Details are given in figure 1A and 2. The haplogroup has likely arisen in the region of the Tibetan plateau [21], thus suggesting that the origin of the individual could have been rather an Asian than European one.

**Figure 3:**
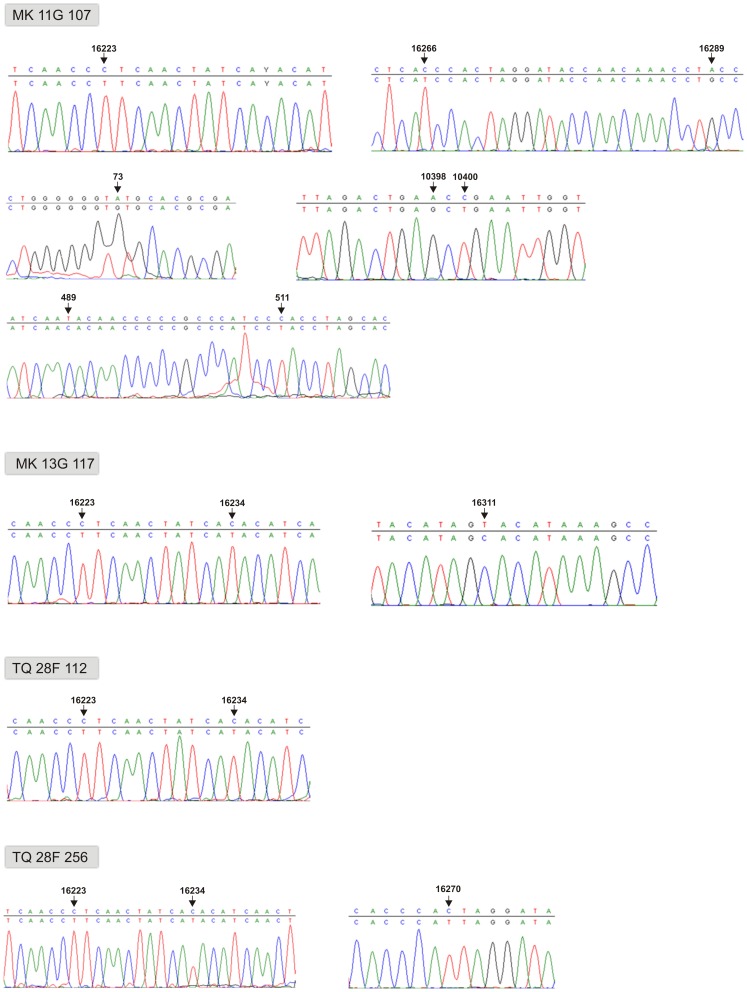
Identified haplotypes of studied individuals as seen from direct sequencing (one of four runs each performed on different portions of pulverized different teeth). HVR-I, HVR-II and coding region of mtDNA for individual MK 11G 107; HVR-I for individuals MK 13G 117, TQ 28F 112 and TQ 28F 256. Obtained consensus sequences have been submitted to GenBank and assigned accession numbers KF462390–KF462396.

**Figure 4:**
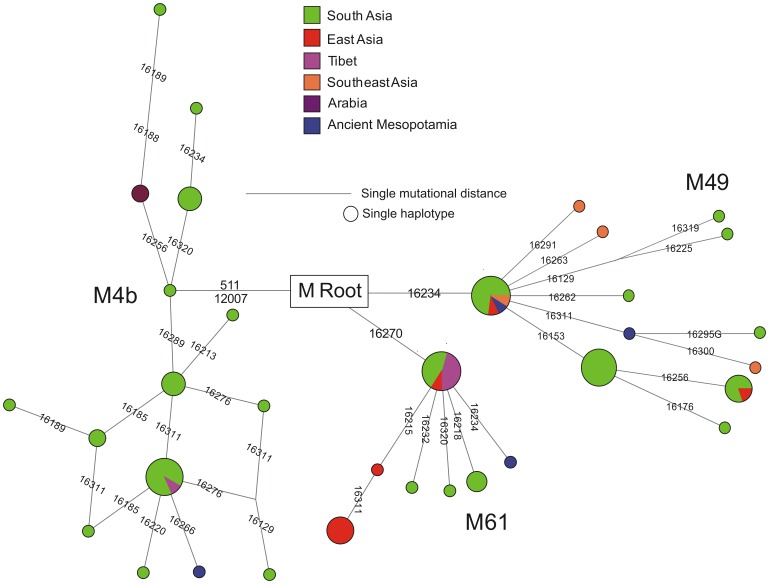
Median joining network [28] of four individuals living in the middle Euphrates valley between 0.2 Kyrs AD and 2.5 Kyrs BC. M–J network of three observed haplogroups (M49, M61 and M4b) shows the distribution of haplotypes among populations from different geographical regions. Circle sizes are proportional to the number of mtDNAs with that haplotype. The data has been taken from published and unpublished sources given in supplementary (Table S3).

The relatively high degree of DNA preservation in the skeleton MK 11G107 inspired a further attempt to isolate DNA from three other specimens, one additional from Tell Masaikh (MK) and two others from Tell Ashara (ancient Terqa, TQ) sites. In contrast to the earlier published haplogroup K found in individual from Terqa [3], an analysis of the HVR-I revealed that individuals TQ 28F 112 and MK 13G 117 belonged to M49 with different haplotypes, and TQ 28F 256 to M49 or M61 (Fig. 3). The identified clades have probably arisen between 25 and 58 Kyrs ago also on the Indian subcontinent, where they still occur [21], [22]. Having sufficiently high access to skeletal material of specimen MK 11G107, a more advanced molecular analysis was undertaken, which showed changes also within HVR-II and the coding region, confirming haplogroup M4b1, presumed earlier only on the basis of the HVR-I sequence (Fig. 1A and 3). An analysis of commonly available databases revealed several haplotypes almost identical with that of MK 11G107, present in people living today in Tibet [23], the Himalayas (Ledakh) [24] and Pakistan [25] (Fig. 1B), and having a reverse mutation at the hot spot 16311 [23] in common with the fossil haplotype [26], [27]. Using median joining network [28] we attempted to draw the four observed haplotypes from those of the extant neighbouring populations (Tab.S3) which confirmed their restriction to the South, East and Southeast Asia regions (Fig. 4). They belong mostly to subclades of South Asian-Tibet specific haplogroups absent today in Mesopotamia [26], [27] (Fig. 3). Only complete sequencing will help to narrow down the geography and indicate precise origin of the studied individuals. However, at this step of the analysis a continuity between Trans-Himalaya and Mesopotamia regions in ancient time is likely which has been broken down as a result of recent population movements. Probably, significant depopulation resulting from the Mongolian invasions of the late 13^th^ century AD [29], followed by repopulation by Bedouin tribes in the 17^th^ century [30] and farmers from southern Anatolia and western Syria during the 19^th^ and 20^th^ century [31] are among the possible factors which may have shifted the gene pool profile of the region.

Assuming the mentioned origin of the identified haplogroups and a result of median joining network analysis, it may be claimed that the studied individuals represent genetic association with the Indian subcontinent. The fact that the studied individuals comprised both males and a female, each living in a different period and representing different haplotypes, suggests that the nature of their presence in Mesopotamia was rather long-lasting than incidental. The close ancestors of specimens TQ 28F 112 and TQ 28F 256 could fall within the population founding Terqa, the historical site constructed probably in the early Bronze Age [1], at time only slightly preceding the dating of the skeletons. All the studied remains could have been also left by descendants of much earlier migration waves spreading clades of macrohaplogroup M from the nearby subcontinent. It cannot be excluded that among them were people involved in the founding of the Mesopotamian civilizations. For instance, it is commonly accepted that the founders of Sumerian civilization came from the outside of the region, their exact origin is, however, still a matter of debate. It is suggested that migrants of Iranian, Indian [32], [33] or even Tibetan affinity [34] founded the Sumerian civilization, which suggestion can be supported by comparing the Tibeto-Burman and Sumerian languages [35]. The migrants could have entered Mesopotamia earlier than 45 centuries ago, during the lifetime of the oldest studied individual, as the Tibetan Plateau was peopled more than 20 Kyrs ago [21], [36]. However, one also should consider the possibility that studied individuals belonged to the groups of itinerant merchants moving along a trade route passing near or through the region, since a recent comparative study of strontium, oxygen, and carbon isotopes content in enamel indicates that people from Indus Valley were present in southern Mesopotamia 3 Kyrs BC [37]. We believe that the identification of mtDNA sequences itself should be acknowledged as significant, leaving its detailed interpretation for further research involving a larger number of specimens, representing other Mesopotamian regions and various periods.

### nuDNA Sequences

**Figure 5:**
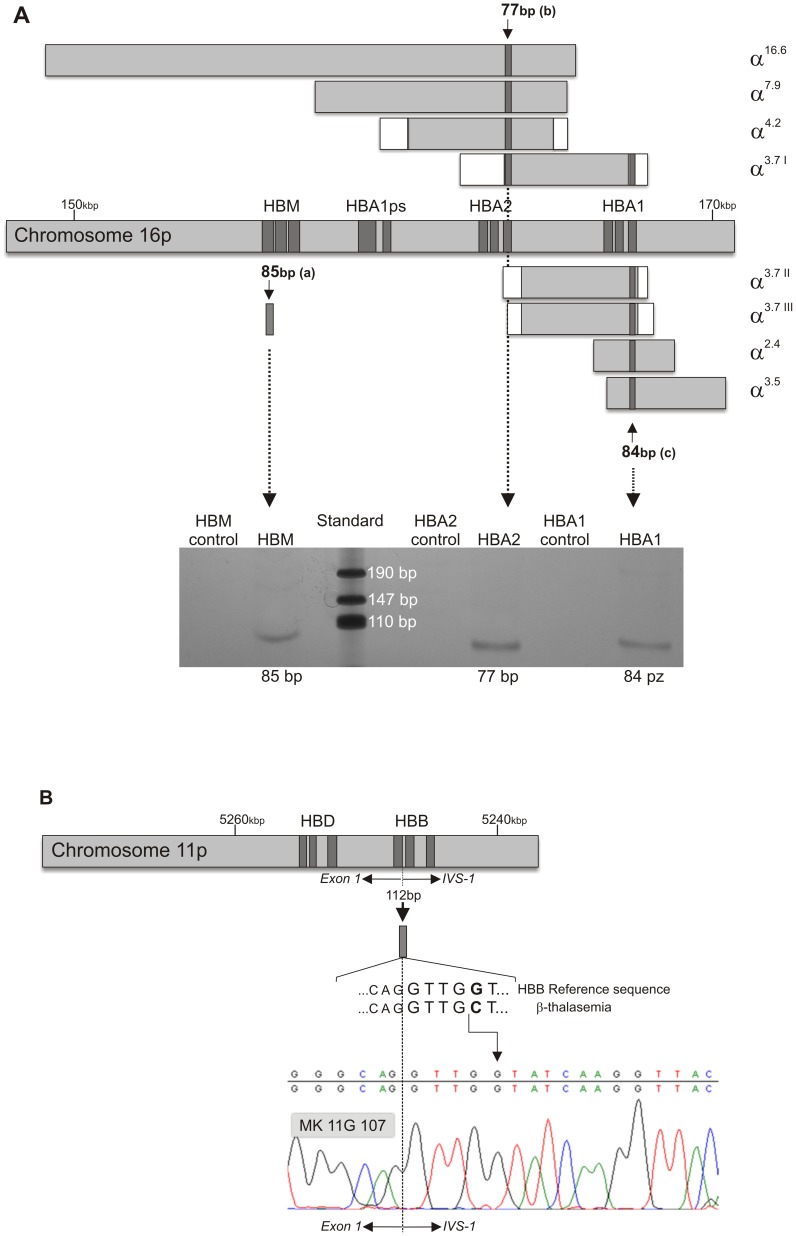
The search for the α and β globin gene cluster mutations in specimen MK 11G 107. The most common ones among today’s inhabitants of the northern part of the Indian subcontinent were identified [39]. **A.** The pattern of identification of the chromosome 16p fragments, deletion of which is responsible for α-thalassemia, shown as filled/unfilled bars indicating regions of certainty/uncertainty of breakpoints. Successful amplification of the *HBM* gene located in close proximity to the region of deletions was used as a positive control, confirming the presence of the thalassemia-predisposing region of the fossil chromosome 16, i.e. the presence of target sequences in the *HBA1* and *HBA2* gene cluster. Although primers were designed to identify 3.7 and 4.2 kb deletion variants, a few others were typed as well. **B.** Pattern of SNP IVS-1 nt 5 G>C identification within the *HBB* gene responsible for β-thalassemia, and the sequenogram of the target region. Primer sequences and PCR conditions are shown in table S1. The size of exons and PCR products does not reflect real proportions of assigned fragments.

The initially analysed nuDNA sequences are responsible for thalassemia and were used to verify the uncertain phenotypic data of the MK 11G 107 specimen. Since the disease can result from numerous types of DNA changes and access to fossil sequences is rather limited, both of which reduce the possibility of identifying the complete spectrum of known mutations leading to thalassemia, only the most common deletions of the *HBA1* and *HBA2* genes and one SNP in the *HBB* were studied. All of them are present today in populations found in the Indian subcontinent. Neither the deletions in the *HBA* genes (α-thalassemia), nor SNP in the *HBB* gene (β-thalassemia) were found. The type of thalassemia mutations studied and the procedure used for their identification are summarized in Tab. S1, Fig. 5a and 5b.

Using relatively well-preserved nuclear sequences, we have attempted to identify other nuclear alleles of significance: *ΔF508 CFTR* (HRM), *LCT-13910T* (sequencing) and *Δ32 CCR5* (PAGE). Neither the pathological *CFTR* allele, the T allele responsible for lactase persistence nor *Δ32 CCR5* were found (Tab. S2).

All nuclear sequences are, to our knowledge, the oldest and the only ones identified until now in samples from Mesopotamian archaeological sites. The above results clearly indicate the possibility of retrieval of Near Eastern fossil DNA if skeletons stayed *post mortem* in favourable conditions (pH of soil and depth of deposition) and DNA isolation is performed shortly after uncovering of the remains [17]. This gives hope for conducting studies at the population level.

## Supporting Information

Table S1 Amplified fragments, sequence of primers and PCR conditions.
(DOCX)

Table S2 Identification of LCT-13910 C/T, ΔF508 CFTR and CCR5 sequences. Sequence of primers and PCR conditions are shown in Table S1. Highlighted in darker-grey represent a comparison of nuclear alleles found in nuDNA of individual MK 11G 107 with those of the only people involved in sampling/anthropological examination and molecular analysis showing the absence of their nuDNA in Mesopotamian sample.
(DOCX)

Table S3 The HVR-I haplotype list of the samples used in NETWORK construction. Region 16110–16340 has been included in the present analysis.
(DOCX)
