# Supplementary material for: mtDNA from the Early Bronze Age to the Roman Period Suggests a Genetic Link between the Indian Subcontinent and Mesopotamian Cradle of Civilization
Source: PLoS One. 2013 Sep 11;8(9):e73682. doi: 10.1371/journal.pone.0073682 (PMC3770703; doi:10.1371/journal.pone.0073682)
Supplement: Table S1 — Amplified fragments, sequence of primers and PCR conditions. (DOCX) [file pone.0073682.s001.docx]

Table S1.

| **Fragment/allele** | **Primer pairs** | **Product length** | **Annealing temp. ^o^C** |
| --- | --- | --- | --- |
| HVR I [[1](#_ENREF_1)] (16095-16262) | CGTACATTACTGCCAGCC TGGTATCCTAGTGGGTGAG | 186 bp | 55 |
| HVR I [[1](#_ENREF_1)] (16232-16370) | CACACATCAACTGCAACTCC TCAAGGGACCCCTATCTGAG | 168 bp | 55 |
| HVR II (47- 176) | GCATTTGGTATTTTCGTCTGG TTGAACGTAGGTGCGATAAAT | 130 bp | 57 |
| HVR II (120- 275) | CGCAGTATCTGTCTTTGATTCC CTGTGTGGAAAGTGGCTGTG | 156 bp | 57 |
| Coding sequence 10345- 10488 | TCATCCTAGCCCTAAGTCTG TTATGTAAATGAGGGGCATT | 144 bp | 55 |
| Coding sequence 398 – 534 | TTTTATCTTTTGGCGGTATG GTTAGCAGCGGTGTGTGT | 137 bp | 57 |
| C/T_-13910_ (rs4988235) | GCGCTGGCAATACAGATAAGATA AATGCAGGGCTCAAAGAACAA | 111 bp | 55 |
| ∆*F508* [[2](#_ENREF_2)] (rs113993960) | GGATTATGCCTGGCACCATTA TTCTAGTTGGCATGCTTTGATGA | 95/92 bp | 61 |
| ∆ *CCR5* (rs333) | CCAGGAATCATCTTTACCAG CAGCCCCAAGATGACTATC | 126/94 bp | 56 |
| HBA1 | AGTTCCTGGCTTCTGTGAGC CCCAAGGGGCAAGAAGCAT | 84 bp | 60 |
| HBA2 | AGTTCCTGGCTTCTGTGAG GGCAGGAGGAACGGCTAC | 77 pz | 60 |
| HBM | AAGTTCCTGACTGGTGTGGC TTGACAGGCACAGACCAAGG | 85 bp | 60 |
| IVS-1 nt 5, G-C (rs33915217) | AGGAGAAGTCTGCCGTTACT TTGGTCTCCTTAAACCTGTC | 112 bp | 56 |

References

1. Fernández E, Ortiz JE, Torres T, Pérez-Pérez A, Gamba C, et al. (2008) Mitochondrial DNA genetic relationships at the ancient Neolithic site of Tell Halula. Forensic Science International: Genetics Supplement Series 1: 271-273.

2. Hummel S, Herrmann B, Rameckers J, Muller D, Sperling K, et al. (1999) Proving the authenticity of ancient DNA by comparative genomic hybridization. Naturwissenschaften 86: 500-503.
