# Supplementary material for: mtDNA from the Early Bronze Age to the Roman Period Suggests a Genetic Link between the Indian Subcontinent and Mesopotamian Cradle of Civilization
Source: PLoS One. 2013 Sep 11;8(9):e73682. doi: 10.1371/journal.pone.0073682 (PMC3770703; doi:10.1371/journal.pone.0073682)
Supplement: Table S2 — Identification of LCT-13910 C/T, ΔF508 CFTR and CCR5 sequences. Sequence of primers and PCR conditions are shown in Table S1. Highlighted in darker-grey represent a comparison of nuclear alleles found in nuDNA of individual MK 11G 107 with those of the only people involved in sampling/anthropological examination and molecular analysis showing the absence of their nuDNA in Mesopotamian sample. (DOCX) [file pone.0073682.s002.docx]

Table S2.

| **Specimen/allele** | ***LCT-13910 C>T*** | ***CFTR/***  ***ΔF508 CFTR*** | ***CCR5/ Δ32 CCR5*** |
| --- | --- | --- | --- |
| MK 11G 107 | C/C | *wild/wild* | *wild/wild* |
| MK 13G 117 | C/C | nd* | - |
| TQ 28F 112 | nd | nd | - |
| TQ 28F 256 | C/C | *wild/wild* | *-* |
| Archaeologist | C/T | *wild/wild* | *wild/wild* |
| Molecular biologist 1 | C/C | *wild/wild* | *wild/ Δ32 CCR5* |
| Molecular biologist 2 | C/T | *wild/wild* | *wild/wild* |

* - not detected
