# Supplementary material for: mtDNA from the Early Bronze Age to the Roman Period Suggests a Genetic Link between the Indian Subcontinent and Mesopotamian Cradle of Civilization
Source: PLoS One. 2013 Sep 11;8(9):e73682. doi: 10.1371/journal.pone.0073682 (PMC3770703; doi:10.1371/journal.pone.0073682)
Supplement: Table S3 — The HVR-I haplotype list of the samples used in NETWORK construction. Region 16110–16340 has been included in the present analysis. (DOCX) [file pone.0073682.s003.docx]

Table S3.

| **Region** | **Id** | **HVS-I (-16000 nt)** | **Haplogroup** | **Reference** |
| --- | --- | --- | --- | --- |
| Myanmar | JX289094 | 223-234 | M49 | GenBank |
| Myanmar | JX289099 | 223-234-300-311 | M49 | GenBank |
| Myanmar | JX289133 | 223-234-291 | M49 | GenBank |
| India | Reddy2007a | 153-223-234 | M49 | Reddy et al. 2007 |
| India | Reddy2007b | 153-176-223-234 | M49 | Reddy et al. 2007 |
| India | Br87 | 129-223-234-319 | M49 | Present study |
| India | Kh004 | 129-223-225-234 | M49 | Present study |
| India | Wa3 | 153-223-234 | M49 | Chandrasekar et al. 2009 |
| India | CSK2009 | 153-223-234-256 | M49 | Chandrasekar et al. 2009 |
| India | SK4 | 153-223-234-256 | M49 | Chandrasekar et al. 2009 |
| China | Monba8 | 153-223-234-256 | M49 | Kong et al. 2011 |
| China | Bai6 | 223-234 | M49 | Kong et al. 2011 |
| Nepal | Nepalese011 | 223-234-295G-311 | M49 | Wang et al. 2012 |
| Nepal | Nepalese185 | 093-223-234 | M49 | Wang et al. 2012 |
| India | D285 | 223-234 | M49 | Present study |
| India | Z015 | 223-234-262 | M49 | Present study |
| Ancient Mesopotamia | TQ28F112 | 223-234 | M49 | Present study |
| Ancient Mesopotamia | MK13G117 | 223-234-311 | M49 | Present study |
| Laos | Laos1 | 93-223-270 | M61 | Dodner et al. 2011 |
| India | CSK2009 | 223-270-362 | M61 | Chandrasekar et al. 2009 |
| Tibet | Tib2840 | 93-270-362 | M61 | Kong et al. 2011 |
| China | Yi334 | 215-223-270-362 | M61 | Kong et al. 2011 |
| Tibet | Tib01F5 | 223-270 | M61 | Peng et al. 2010 |
| China | Naxi04 | 86-215-223-270-311 | M61 | Peng et al. 2010 |
| China | Da18 | 215-223-270-311 | M61 | Peng et al. 2010 |
| Tibet | 158chaun | 223-270 | M61 | Qin et al. 2010 |
| Tibet | 5434 | 223-270-362 | M61 | Qin et al. 2010 |
| Tibet | 5567 | 223-270-362 | M61 | Qin et al. 2010 |
| Tibet | 174 | 223-270-320 | M61 | Qin et al. 2010 |
| Tibet | A306 | 223-232-270 | M61 | Qin et al. 2010 |
| India | TAP | 86-218-223-270 | M61 | Fornarino et al. 2009 |
| Ancient Mesopotamia | TQ28F256 | 223-234-270 | M61 | Present study |
| Saudi Arabia | 633 | 223-256-311 | M4b | Abu-Amero et al. 2008 |
| Saudi Arabia | 634 | 223-256-311 | M4b | Abu-Amero et al. 2008 |
| India | A9 | 223-311 | M4b | Sun et al. 2006 |
| India | A39 | 223-289-311 | M4b | Sun et al. 2006 |
| India | A45 | 213-223-289-311 | M4b | Sun et al. 2006 |
| India | KU4 | 93-223-311-320 | M4b | Chandrasekar et al. 2009 |
| India | X3 | 223-289-311 | M4b | Thanseem et al. 2006 |
| Sri-Lanka | Lob2 | 185-223-289-311 | M4b | Kivisild et al. 1999 |
| Sri-Lanka | 27 | 185-189-223-289-311 | M4b | Metspalu et al. 2004 |
| India | M12 | 188-189-223-256-311 | M4b | Bamshad et al. 1998 |
| India | WB | 223-234-311-320 | M4b | Basu et al. 2003 |
| India | UN | 223-289 | M4b | Present study |
| Nepal | Napalese055 | 223-276-289-311 | M4b | Wang et al. 2012 |
| Tibet | Tib557 | 92-223-289 | M4b | Kong et al. 2011 |
| Pakistan | Pk121 | 129-223-276-289 | M4b | Alla Rakha et al. 2010 |
| Pakistan | Pk80 | 185-223-289 | M4b | Alla Rakha et al. 2010 |
| Pakistan | Pk158 | 220-223-289 | M4b | Alla Rakha et al. 2010 |
| India | BS48 | 223-289 | M4b | GenBank |
| Ancient Mesopotamia | MK11G107 | 223-266-289 | M4b | Present study |

References
